# Interferon-Induced Genes of the Expanded IFIT Family Show Conserved Antiviral Activities in Non-Mammalian Species

**DOI:** 10.1371/journal.pone.0100015

**Published:** 2014-06-20

**Authors:** Mónica Varela, Patricia Diaz-Rosales, Patricia Pereiro, Gabriel Forn-Cuní, Maria M. Costa, Sonia Dios, Alejandro Romero, Antonio Figueras, Beatriz Novoa

**Affiliations:** Instituto de Investigaciones Marinas (IIM), CSIC, Vigo, Spain; University of Berne, Switzerland

## Abstract

Interferon-induced proteins with tetratricopeptide repeats (IFITs) are involved in the protective response to viral infection, although the precise mechanism of IFITs for reducing viral proliferation is currently unknown. The interaction with the translation initiation factor eIF-3 or viral proteins and the sequestering of viral RNA have been proposed as potential antiviral functions for these proteins. In humans, four members of this family have been characterized. Nevertheless, information about these proteins in fish is almost non-existent. Exploiting the conservation of synteny between human and zebrafish genomes, we have identified ten members of the IFIT family located on four different chromosomes. The induction of these genes was examined both *in vitro* and *in vivo* after interferon (IFN) administration and rhabdovirus challenge. Whereas an induction of IFIT genes was observed after interferon treatments (IFNΦ1, IFNΦ2 and IFNΦ3), the viral infection did not affect these IFN-induced genes *in vitro*, and even reduced the IFN-induced expression of these genes. The response was largely different *in vivo*, with a broad up-regulation of IFIT genes after viral challenge. In addition, three selected IFITs were cloned in an expression vector and microinjected into zebrafish larvae to examine the protective effect of IFITs upon viral infection. Reduction in the mortality rate was observed confirming a conserved antiviral function in non-mammalian species.

## Introduction

Host antiviral innate immune responses begin with the detection of viruses, which triggers the induction of cellular and molecular effectors with broad antiviral activities [1], including type I interferon (IFN) and hundreds of IFN-stimulated genes (ISGs), which contribute to the overall effects against a given virus [2], [3].

In fish, although numerous IFN genes have been characterized (reviewed in [4]), their classification is controversial, as these genes are more diverse than previously thought, and their genomic structures, containing introns, are reminiscent of mammalian type III interferons (IFN-λ), although the encoded proteins are similar to type I interferons [5]. Fish type I IFNs, named as interferon-phi (IFNΦ) were classified in two groups: group I (comprising IFNΦ1 and IFNΦ4) and group II (composed by IFNΦ2 and IFNΦ3) [6], [7]. These two groups of IFNs do not bind to the same receptor complexes in zebrafish, as was shown by Aggad et al. [8].

Knowledge of the antiviral properties of individual ISGs is mostly limited to a few intensively studied examples, such as PKR [9] or MX [10], [11]. MX is a well-studied ISG in fish but our knowledge of other ISGs (e.g., Vig-1/viperin, ISG15, finTRIMs, and PKR) in fish is limited (reviewed in [12]). Even in mammals, although the importance of the IFN system is clear, there is not enough information concerning the mechanism underlying the IFN-mediated inhibition of viral replication, particularly *in vivo* [13].

Among ISGs, a protein family called IFIT (interferon-induced proteins with tetratricopeptide repeats), which is characterized by tetratricopeptide repeats (TPR domains), has been examined in higher vertebrates [14]–[16]. Recent studies have shown that the IFIT family is conserved in mammals, amphibians and birds, but these proteins are not present in yeast, plants or lower animals, such as fruit fly and nematodes [16]. The members of this protein family were initially named according to their molecular weights (ISG54/P54, ISG56/P56, ISG58/P58 and ISG60/P60), although currently the most relevant and recent publications have adopted the term IFIT [16]. IFIT proteins are involved in many processes in response to viral infection and other functions, such as protein-protein and protein-RNA interactions, double-stranded RNA signaling, cell migration, and proliferation [14], [17]. The transcriptional induction of the IFIT family genes has been described after infection with both DNA- and RNA-viruses [18]–[21] and after bacterial stimulation in a type I IFN-dependent manner [22]–[24]. Although their antiviral mechanisms are still poorly understood, studies have shown that IFIT genes restrict virus replication through the alteration and suppression of protein synthesis or direct binding and sequestering of viral RNA, thereby reducing their infectivity [16].

In fish, information concerning IFIT genes is almost non-existent. Only a few partial or unconfirmed sequences of IFIT genes have been identified using sequencing analyses [16], [25]. A formal characterization of IFIT genes and an in depth study of their regulation under different stimuli has never been done in fish.

Teleosts fish offer an interesting model for the study of IFITs, not only for the clear interest in this ISG family in relation to viral infection, which constitutes an important threat, particularly for cultured fish, but also due to the ancient separation of fish from tetrapods and the great diversity of fish species. In addition, the advantage of the increased use of zebrafish (*Danio rerio*) as an important vertebrate model for studies in developmental and biomedical research, hematopoiesis and recently, immunology, has facilitated the development of genomic tools that allow the identification of new gene families. In the present work, we describe the complete repertoire of IFIT genes in zebrafish. Our study reveals a protein family forged through ancient duplication events, according with the results recently published [25]. To further explore the antiviral properties of these IFN-stimulated genes, *in vivo* and *in vitro* experiments were conducted in zebrafish after treatment with different recombinant IFNsΦ and after viral infection. Moreover, the protective effect of three selected zebrafish IFITs upon viral challenge was also examined *in vivo*.

## Results

### Defining the Complete Repertoire of IFIT Genes in Zebrafish

**Figure 1:**
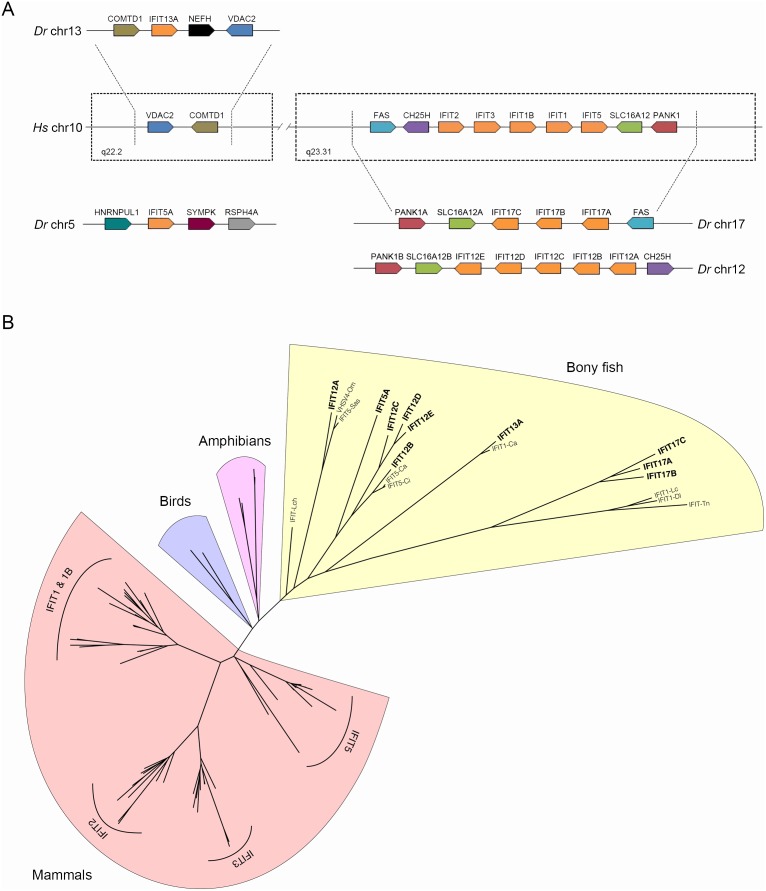
Synteny and phylogenetic analysis. A. Comparative gene location of IFIT sequences between human chromosome 10 and zebrafish chromosomes 5, 12, 13 and 17. The genes flanking the IFIT cluster, located at the q23.31 region of human chromosome 10, showed a high degree of synteny with the zebrafish chromosomes 17 and 12. Moreover, the genes surrounding zebrafish IFIT 13 were located on the same human chromosome but in the q22.2 region. An IFIT sequence was also present on zebrafish chromosome 5. B. Phylogenetic relationship between the IFIT proteins from bony fish and similar proteins described in other vertebrates, such as amphibians, birds and mammals. Lch: *Latimeria chalumnae*; Om: *Oncorhynchus mykiss*; Sas: *Salmo salar*; Ca: *Carassius auratus*; Ci: *Ctenopharyngodon idella*; Lc: *Larimichthys crocea*; Dl: *Dicentrarchus labrax*; Tn: *Tetraodon nigroviridis*.

Using a zebrafish genome-wide blast search, we detected a high degree of synteny between the human chromosome 10 (region q23.31) and the zebrafish chromosomes 17 and 12 (Figure 1A). Our analysis confirmed the presence of five and three IFIT genes on zebrafish chromosomes 12 and 17, respectively. Moreover, another two genes were identified as similar to IFITs (one gene on chromosome 5 and the other gene on chromosome 13).

The zebrafish IFIT sequences were named according to their chromosomal position: IFIT5A (ENSDARG00000088069), IFIT12A (ENSDARG00000008098), IFIT12B (ENSDARG00000007467), IFIT12C (ENSDARG00000090537), IFIT12D (not identified in the Ensembl database), IFIT12E (ENSDARG00000090977), IFIT13A (ENSDARG00000057173), IFIT17A (ENSDARG00000071012), IFIT17B (ENSDARG00000043584) and IFIT17C (ENSDARG00000056976). In order to confirm the IFIT sequences, we designed specific primers to amplify and sequence the 10 zebrafish IFIT genes (primers in Table S1). The confirmed full-length ORFs were submitted to GenBank under accession numbers KF418356–KF418365.

The block of human IFIT genes and the pseudogene IFIT1B (IFIT-1, 1B, 2, 3 and 5) is flanked downstream by the SLC16A12 (solute carrier family 16, member 12) and PANK1 (pantothenate kinase 1) genes. These genes are also present on zebrafish chromosomes 17 and 12 but are situated upstream of the IFIT region (Figure 1A). The FAS (TNF receptor superfamily, member 6) and CH25H (cholesterol 25-hydroxylase) genes are located upstream of the human IFIT cluster and showed sub-partitioning in zebrafish. Thus, FAS is located on chromosome 17 and CH25H is located on chromosome 12, and both genes have an inverted orientation at the 3′ region of the IFIT genes (Figure 1A). The q22.2 region of human chromosome 10 also showed homology with zebrafish chromosome 13. One IFIT-related gene (IFIT13A) was identified between the COMTD1 (catechol-O-methyltransferase domain containing 1) gene at the 5′ end and the NEFH (neurofilament, heavy polypeptide) and VDAC2 (voltage-dependent anion channel 2) genes at the 3′ end of this chromosome. In this case, synteny was not conserved because there were no IFIT genes between the VDAC2 and COMTD1 genes in human chromosome 10 (Figure 1A). Moreover, another IFIT gene was identified on chromosome 5, but it was not possible to identify a conserved region between both species (Figure 1A).

### Phylogenetic Tree and Analysis of Darwinian Selection

Data mining for IFIT protein sequences retrieved 77 different sequences, 60 of which belonged to mammalian species, 2 bird species, 6 amphibian species and 9 fish species (Table S2). We used these sequences to construct a phylogenetic tree, revealing a clear separation of the IFIT sequences among the analyzed taxonomical classes (Figure 1B). The tree topology categorized the mammalian IFIT sequences into four main branches, IFIT1/1B, IFIT2, IFIT3 and IFIT5 homology groups, with great confidence values; however, the phylogenetic relationship among non-mammalian IFIT sequences was not clear, potentially reflecting the minor representation of these sequences in the entire analysis. For example, the evolutionary relationship of the different IFIT genes across fish species was not confidently resolved, although some sequences were orthologous. However, an interesting pattern emerged, branching the zebrafish IFIT sequences belonging to the same chromosome.

We estimated the dN/dS ratios (ω) among the zebrafish IFIT sequences to quantify the selection pressure acting on IFITs genes and determined that the genes located on chromosome 17 underwent positive Darwinian selection (ω>1). The dN/dS ratio observed between IFIT17A and IFIT17B was 1.2928, whereas this value was higher between IFITs 17B and 17C (ω = 1.4667) and between IFITs 17A and 17C (ω = 1.5130). The IFIT genes located on chromosomes 5, 12 and 13 were not subjected to this evolutionary mechanism, obtaining ω values lower than 1.

### Sequencing Analysis and Structure Domains

**Figure 2:**
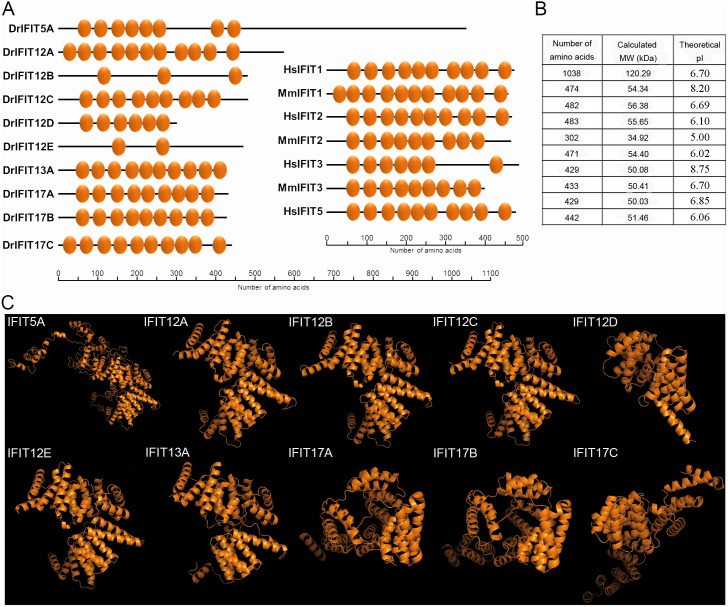
Sequence analysis and structural domain of the zebrafish IFITs. A. Tetratricopeptide repeat (TPR) motifs on the 10 IFIT proteins present in zebrafish. Orthologous proteins from human and mouse are shown for comparative purposes. B. Length, calculated molecular weight (kDa) and theoretical pI of the different zebrafish IFITs. C. 3D-structure of zebrafish IFITs, predicted using I-TASSER server, selecting the model with the best C-score and viewed using PyMOL.

The study of the domain structure revealed the presence of the characteristic TPR motifs in all the analyzed sequences (Figure 2A), but the number of these repeats was variable, ranging from two up to eleven repetitions. However, we also observed variability in the number and position of these characteristic domains in the four human genes and in the three IFITs described in mice (Figure 2A). The values of identity and similarity between zebrafish and human and murine proteins were lower than 37% and 62%, respectively (Table S3). The number of amino acids varied between 302 and 483 residues for all sequences, except for isoform 5A, which presented a total of 1038 amino acids. Thus, differences in calculated molecular weights were also observed, whereas most of the proteins presented molecular weights ranging from 50–56 kDa, the 12D isoform was 34.92 kDa and IFIT5A was 120.29 kDa (Figure 2B). Regarding the theoretical isoelectric point (pI), the isoforms located on chromosomes 5, 12 and 17 presented values lower than 7.0, except 12A, whose pI value was 8.20. IFIT13A also showed a more basic pI value of 8.75 (Figure. 2B).

We also examined the tridimensional structure of the IFITs and identified three different structural models (Figure 2C). The TM-scores observed for zebrafish IFITs revealed that human IFIT5 was the main analog protein for IFIT5A (TM-score = 0.448), IFIT12A (TM-score = 0.981), IFIT12B (TM-score = 0.966), IFIT 12C (TM-score = 0.951), IFIT12D (TM-score = 0.950), IFIT12E (TM-score = 0.969) and IFIT13A (TM-score = 0.952). However, human ISG54 or IFIT2 was the best analog for IFIT17A (TM-score = 0.947) and IFIT17B (TM-score = 0.943), and interestingly, the superhelical TPR-repeat domain of O-linked GlcNAc transferase was the template for the construction of the IFIT 17C 3D-structure (TM-score = 0.813).

### Constitutive and Tissue-specific Expression of IFIT Genes

**Figure 3:**
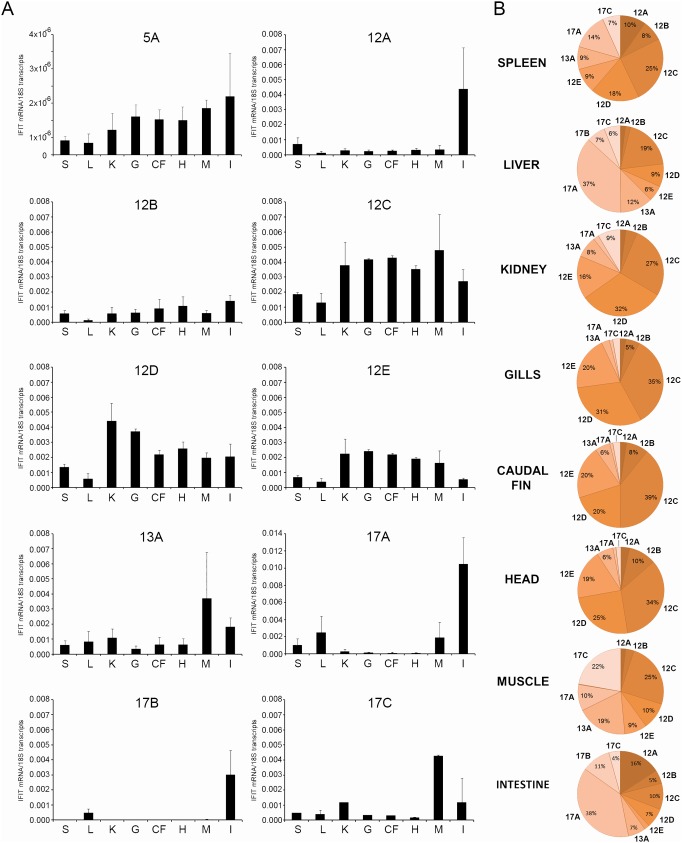
Tissue-specific expression of zebrafish IFIT genes. A. Constitutive expression of IFIT genes in tissues of adult zebrafish (S: Spleen; L: Liver; K: Kidney; G: Gill; CF: Caudal fin; H: Head; M: Muscle; I: Intestine). For basal expression of each IFIT form, tissues were sampled and pooled, yielding a total of 4 pools of 5 fish per organ. The relative expression level of each gene, normalized to the expression level of the 18 S ribosomal RNA gene in the same tissue, was expressed as arbitrary units. The graphs represent the mean ± standard error of 4 independent samples. B. Relative proportion of the IFIT transcripts in different zebrafish tissues.

The analysis of 8 different adult zebrafish tissues revealed a higher basal expression of IFIT genes on chromosome 12, being IFIT12C the gene with the largest presence in the whole of the tissues analyzed (spleen, kidney, gill, caudal fin and head). Interestingly, IFITs with low expression in most of the tissues, showed significantly higher expression in the intestine (12A, 17A, 17B) or muscle (13A, 17C) (Figure 3A). Regarding to the relative proportion of the IFIT genes in the analyzed tissues, whereas in spleen, kidney, muscle, intestine and liver all the IFITs were present, in gills IFITs from chromosome 12 were predominant (Figure 3B).

The constitutive expression of IFIT genes was also analyzed in ZF4 and kidney primary cells (Figure S1). ZF4 cells presented higher expression levels than kidney primary cell cultures. In addition, IFITs from zebrafish chromosome 12 showed a higher basal expression in both cell types than those from chromosomes 5, 13 or 17, except for isoform 12B, which showed lower expression in ZF4 cells.

### Interferons Induce the Expression of IFIT Genes *In*?*vitro*

First, we determined whether three selected zebrafish interferons (IFNΦ1, IFNΦ2 and IFNΦ3) induced the expression of zebrafish IFITs. Therefore, we analyzed the biological activity of recombinant zebrafish IFNsΦ. The activity of supernatant from HEK-293 cells transfected with plasmids containing IFNs sequences was first confirmed by a decrease in the viral titer of spring viraemia of carp virus (SVCV) and the induction of MXab (isoforms a and b) expression in ZF4 and kidney primary cell culture, as shown in the Figure S2A and S2B.

**Figure 4:**
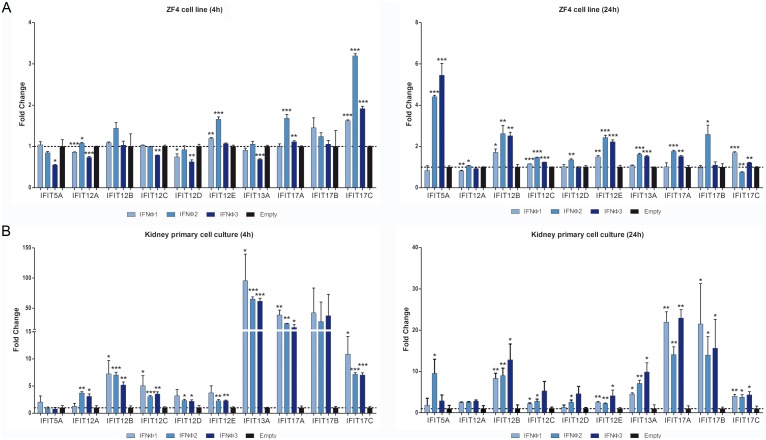
*In vitro* effect of IFNs on IFITs expression in ZF4 cells and kidney primary cell cultures. A. Expression levels of IFIT genes in ZF4 cells after 4 and 24 hours of stimulation with supernatants from transfected HEK-293 cells with plasmids containing sequences for zf-IFNΦ1, zf-IFNΦ2 and zf-IFNΦ3. B. Kidney primary cell cultures after 4 and 24 hours of stimulation with supernatants from transfected HEK-293 cells with plasmids containing sequences for zf-IFNΦ1, zf-IFNΦ2 and zf-IFNΦ3. After 4 and 24 hours of stimulation, RNA was extracted and the cDNA was synthesized. Analysis of the gene expression was performed through real-time PCR, using 18 S ribosomal RNA as a housekeeping gene. The expression level of each gene was expressed as fold-change with respect to the empty plasmid group. The data are represented as the mean ± standard error of three independent samples. Significant differences among cells transfected with IFNΦ plasmids and empty plasmid were displayed as ***(0.0001<p<0.001), **(0.001<p<0.01) or *(0.01<p<0.05).

The treatment of ZF4 cells and kidney cell cultures with interferons induced changes in the expression of IFIT genes. Overall, the results showed higher expression values for all IFIT genes in kidney cells than in ZF4 cells (Figure 4A and 4B). As expected, these results suggest a role of IFNs in the induction of zebrafish IFITs. Moreover, the fact that IFITs basal expression was higher in ZF4 than in primary cell cultures and that these cells showed a higher response to IFNs suggest a cell-specific response or mechanism.

In ZF4 cells, treatment with IFNs after 4 hours did not induce high levels of IFITs expression, with the exception of IFIT17C, which was significantly induced, regardless of the IFNΦ used. Interestingly, a significant down-regulation of isoforms 5A, 12A, 12C, 12D and 13A was observed in cells treated with the IFNΦ3 (Figure 4A). A higher number of isoforms was significantly induced at 24 hours compared with the results observed at 4 hours, and the IFIT on chromosome 5 was the most induced through IFNΦ2 and IFNΦ3. The IFITs on chromosome 12 (12B, 12C and 12E) were significantly induced, regardless of the IFNΦ used (Figure 4A).

In primary cell culture, the IFIT genes located on chromosomes 13 and 17 were more induced than those on chromosomes 5 and 12, and isoform 13A showed the strongest induction at 4 hours (Figure 4B). Regarding the effect of the different IFNsΦ, most of the IFITs on chromosomes 12 and 13 were induced through IFNΦ2 and IFNΦ3 at 4 hours (Figure 4B). Thus, in primary cell culture, only IFIT12B, IFIT12C, IFIT13A, IFIT17A and IFIT17C were significantly induced through IFNΦ1 after 4 hours, whereas, after 24 hours, the significant induction of 12B, 12C, 12E, 13A, 17A, 17B and 17C was observed.

### Modulation of IFITs Expression upon Viral Challenge

**Figure 5:**
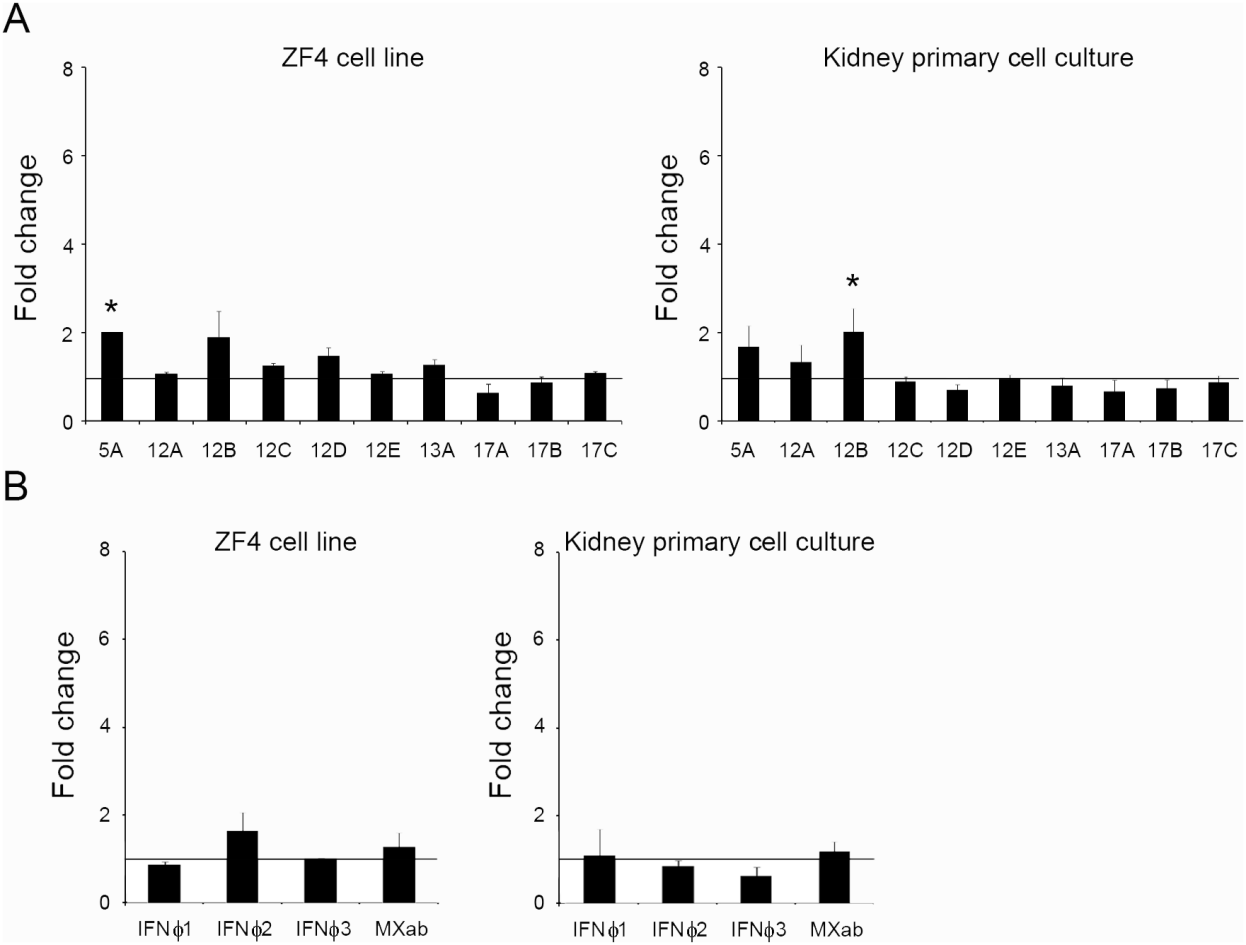
*In vitro* effect of viral infection in ZF4 cells and kidney primary cell cultures. A. Expression of IFIT genes in ZF4 cells and kidney primary cell culture at 24 hours after infection with SVCV. B. MXab and IFN**Φ**1, 2 and 3 expression in ZF4 cells and kidney primary cell culture at 24 hours after infection with SVCV. After 24 hours of stimulation, the RNA was extracted, and the cDNA was synthesized. The analysis of gene expression was performed through real-time PCR, using 18 S ribosomal RNA as housekeeping gene. The expression level of each gene was expressed as fold-change with respect to the control group, non-infected cells. The data are shown as the mean ± error of three independent biological samples. Significant differences among infected and uninfected group were displayed as ***(0.0001<p<0.001), **(0.001<p<0.01) or *(0.01<p<0.05).

Once we determined that zebrafish IFITs were modulated through interferons and showed different tissue expression profiles, we evaluated the effect of an *in vitro* viral infection on the expression of IFITs in ZF4 and kidney primary cells. Surprisingly, SVCV did not modify the expression of the different IFITs (with the exception of IFIT5A in ZF4 and IFIT12B in kidney cells that showed a slight expression increase) (Figure 5A). To determine whether this effect was induced through a direct effect of the virus on IFITs expression or if the virus was affecting the interferon signaling cascade, we measured the expression levels of IFNΦ1, 2 and 3 and the interferon-induced protein MXab after *in vitro* infection. The results showed that IFNΦ1, 2 and 3 and MXab were not induced in either ZF4 or kidney cells through a 24 hours *in vitro* infection (Figure 5B).

**Figure 6:**
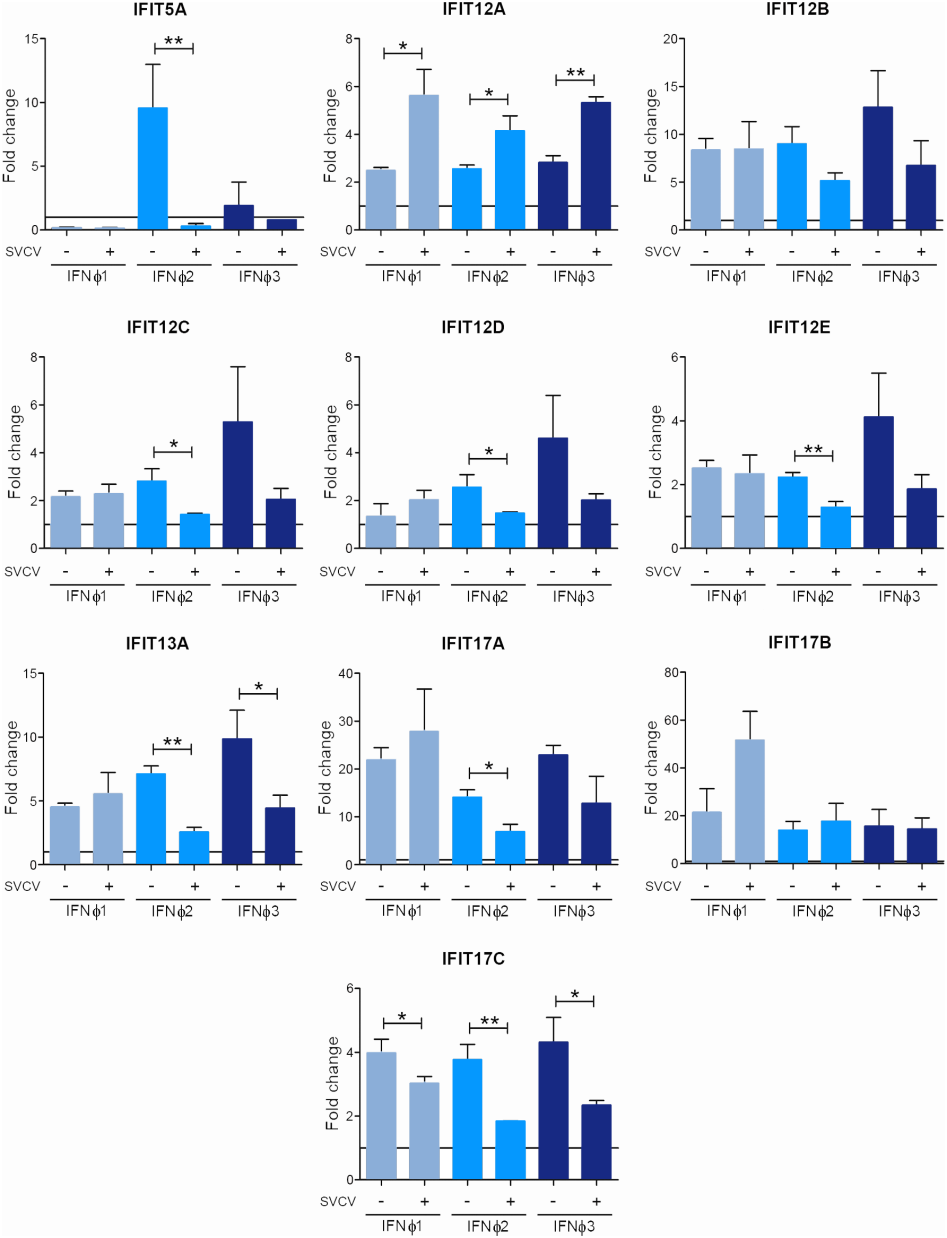
*In vitro* effect of viral infection and IFN treatment in kidney primary cell cultures. Expression level of IFIT genes in kidney primary cell cultures after 24 hours of stimulation with supernatants from transfected HEK-293 cells with plasmids containing sequences for zf-IFNΦ1, zf-IFNΦ2 and zf-IFNΦ3 in combination with SVCV. After 24 hours of stimulation, the RNA was extracted, and the cDNA synthesized. The analysis of gene expression was performed through real-time PCR, using 18 S ribosomal RNA as a housekeeping gene. The effect of the virus infection on the expression of IFITs induced by the different IFNs was represented as a fold-change with respect to the group stimulated with supernatant from cells transfected with the empty plasmid. The data are represented as the mean ± standard error of three independent samples. The asterisks denote significant differences between infected and non-infected groups. Significant differences were displayed as ***(0.0001<p<0.001), **(0.001<p<0.01) or *(0.01<p<0.05).

Next, we determined whether the IFNΦ-induced expression of IFIT genes was also modulated through viral infection. Thus, the effect of the virus on kidney primary cells treated with the recombinant IFNsΦ was analyzed at 24 hours after infection (Figure 6). In general, the virus reduced the expression of interferon-induced IFITs. However, the expression induced through recombinant IFNΦ1 did not show this clear decrease, and in the case of isoforms 12A, an increment in expression was observed after viral infection. In addition, isoform 12A was the only gene that experienced an up-modulation after stimulation with the three recombinant IFNsΦ and the infection with the virus (Figure 6).

**Figure 7:**
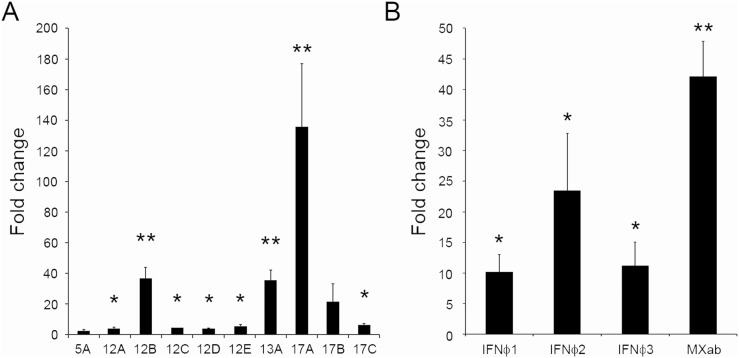
*In vivo* effect of viral infection on IFNs, MXab and IFIT genes expression in kidney. A. Expression of IFIT genes in kidney cells from adult zebrafish at 24 hours after infection with SVCV. B. Expression of IFNs and MXab in kidney cells from adult zebrafish at 24 hours after infection with SVCV. Adult individuals were injected intraperitoneally with 10 µl of SVCV (2.7×10^6^ TCID_50_/ml). RNA was isolated from head kidney cells, 24 hours post-infection. cDNA was obtained, and real-time PCR was performed using 18 S ribosomal RNA as a housekeeping gene. The expression level of each gene was expressed as fold-change with respect to the levels detected in the control group (injected with culture medium). The data are shown as the mean ± standard error of three individuals. The asterisks denote statistically significant differences with respect to the control group. Significant differences were displayed as ***(0.0001<p<0.001), **(0.001<p<0.01) or *(0.01<p<0.05).

To determine whether the trend of down-modulation of IFN-induced genes through SVCV infection also occurred *in vivo*, we examined IFIT genes expression in kidney cells from adult animals injected with the virus. In this case, all the IFITs showed an increase in expression after 24 hours (the increased expression of 5A and 17B were not statistically significant), and 17A showed the highest fold change (Figure 7A). As expected, an up-regulation of all analyzed IFNΦs was observed, being the expression of IFNΦ2 the highest detected. The interferon inducible protein MXab showed a statistically significant increase of 40 fold after the stimulation with SVCV (Figure 7B). These results denoted the different response to the virus after an *in?vitro* or *in?vivo* infection.

### Evaluation of Antiviral Activity of IFIT Genes in Zebrafish Larvae

**Figure 8:**
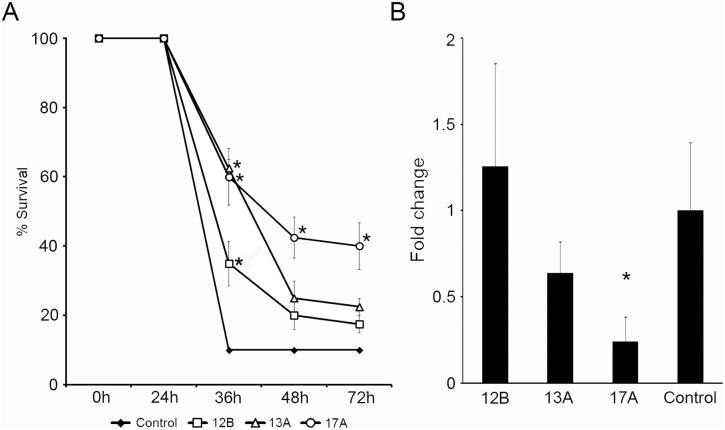
Antiviral activity of IFITs 12B, 13A and 17A in larvae infected with SVCV. A. Antiviral activity of selected IFITs was evaluated in zebrafish larvae. One-cell stage zebrafish embryos were microinjected with 100 pg/egg (final volume of 2 nl) of the recombinant plasmids pcDNA 3.1-IFIT12B, pcDNA 3.1-IFIT13A, pcDNA 3.1-IFIT17A as well as pcDNA 3.1-empty. Three days after plasmid administration, the larvae were microinjected in the duct of Cuvier with 2 nL of a SVCV suspension at a final concentration of 10^3^ TCID_50_/ml. The data are shown as the percentage of survival observed at 3 days after infection. Significant differences (*P*≤0.05) in the percentage of survival between larvae treated with the IFITs and the control group are indicated with asterisks. The results are represented as the mean ± standard error of four independent samples. B. The relative expression level of the viral N gene was analyzed through qPCR at 9 hours post-challenge. The raw data were normalized using the 18 S ribosomal RNA as a housekeeping gene. The results are presented as the mean ± standard error of three biological replicates. Significant differences were displayed as ***(0.0001<p<0.001), **(0.001<p<0.01) or *(0.01<p<0.05).

Next, we analyzed the *in vivo* antiviral activity of selected IFITs in zebrafish larvae previously microinjected with expression vectors containing either IFIT12B, 13A or 17A, followed by infection with SVCV. When zebrafish larvae were infected with SVCV (control group), the mortalities reached the maximum level at 36 hours after challenge, and only 10% of the animals survived the infection (Figure 8A). At this point, the percentages of survival in animals treated with expression vectors containing IFIT sequences were higher than those obtained in the control group. Animals treated with 12B, 13A and 17A showed a final % survival of 17.5, 22.5 and 40%, respectively. At 36?h post-infection, significant differences in the survival were observed in animals treated with the three IFITs, whereas at 48 and 72 hours only the larvae treated with IFIT17A showed a significant increase in survival with respect to the control group.

The transcription of the SVCV N gene was also measured through qPCR at 9 h after infection to determine the effect of these IFITs on viral transcription. At this time point, only larvae injected with the IFIT17A plasmid showed a significant lower viral N gene transcription compared with the infected control group (Figure 8B).

## Discussion

IFITs are a novel IFN-stimulated gene family with antiviral properties not formally described in fish until a recent published work [25]. Using genome synteny and sequence comparison, we identified 10 sequences in the zebrafish genome with homology to human IFIT genes located in chromosome 10 (four genes and one pseudogene). With the exception of IFIT5A, all zebrafish IFIT genes presented similar length and conserved domains.

In contrast to humans, most of the IFIT family genes in zebrafish are located in two chromosomes (12 and 17), similar to the structure observed in dogs [14], [16]. Five IFIT genes were clustered on chromosome 12, as previously described [16], [25], but our analysis also registered the presence of three IFIT genes on chromosome 17, one gene on chromosome 13 and another additional gene on chromosome 5, in agreement with that recently reported [25]. The IFIT genes on chromosomes 5 and 13 did not conserve the synteny with vertebrate chromosomes, likely reflecting genomic translocations [26], [27]. Genome duplication events are powerful drivers of evolution [28], [29], as they provide opportunities for the modification or mutation of the gene duplicates, while critical functions are maintained through the other copies. The modified or mutated duplicated genes might acquire new functions or divide the original functions of the ancestral gene among different isoforms. The presence of different members of the IFIT family genes in fish might facilitate the expansion of innate immune recognition or modulate innate and adaptive immune responses to specific challenges.

The analysis of Darwinian selection was conducted to quantify the selection pressures acting on IFIT genes [30], [31], and the results showed that the genes located on chromosome 17 underwent positive Darwinian selection. This result, together with the phylogenetic analysis, reflected the increased accumulation of evolutionary changes in these genes with respect to the other IFIT genes. The accelerated evolution of the IFIT genes on chromosome 17 might be associated with the direct interaction of these proteins with pathogenic viruses, as an elevated selective pressure and rapid evolution of immune-related genes, particularly those that directly interact with pathogens, was observed compared with non-immune genes [32]–[41].

Phylogenetic analysis confirmed that mammal IFITs are clustered in a main group comprising the four IFIT genes previously described [14], [16]. The sequences from other vertebrates constituted different clusters, depending on the class (amphibians, birds or bony fish), as previously reported [16] however, the construction of an internal classification of the IFIT genes in fish is difficult due to the scarce information available in public databases for other fish species. A deeper analysis of this gene family for organisms belonging to different taxa would help elucidate the evolutionary process involved.

The functional activity of the novel IFIT family described in zebrafish was explored using *in vitro* and *in vivo* experimental models. Most mammalian cell types do not express IFIT genes under basal conditions [15], [42]; however, we observed a constitutive expression of the IFITs particularly the genes located on chromosome 12. In addition, the distribution of the ten different IFIT transcripts in zebrafish displayed distinct patterns of preferential expression at tissue level, and revealed an extremely higher functional complexity than that previously reported in mice [14], [20], [43], [44]. Constitutively expressed genes, such as 12C, 12D and 12E, were present in all tissues, but the low expression of other genes, such as 12B, 13A and 17A, might suggest they are inducible genes. Moreover, the high expression levels observed in the intestine might suggest a specific function for IFIT12A, 17A and 17B in this tissue. Indeed, the IFITs on chromosome 17 are primarily expressed in the liver and intestine. These results suggest that IFIT genes might have non-redundant antiviral functions, as previously suggested in mice [20], [44], reflecting the differentiation and subsequent specialization of the members, potentially facilitated through the gene expansion observed in fish.

The expression of IFIT genes was analyzed in kidney primary cell cultures and in ZF4 cells in response to an IFNsΦ treatment. Recombinant zebrafish IFNsΦ (1, 2 and 3) significantly reduced the viral titer in ZF4 cells infected with SVCV, as previously reported [45], and induced a rapid and high expression of the interferon-inducible MXab genes in kidney primary cell cultures and ZF4 cells, as described in other fish models. Interestingly, the IFNsΦ from group II (IFNΦ2 and 3) showed higher antiviral activity and induction of the MXab genes than that observed for IFNsΦ from group I (IFNΦ1). This differential antiviral activity observed between IFNs from group I (IFNΦ1) and group II (IFNΦ2 and 3) could reflect the induction of several response pathways, as these molecules do not bind the same receptor complexes [8]. The treatment of cells with IFNsΦ also induced a rapid increase in IFIT genes expression in kidney cells (mainly IFIT13A and 17s) consistent with previous studies [16]. The modulation of IFIT genes in ZF4 cells was much lower than that observed in kidney cells most likely because ZF4 is not an immune cell line [46], and the effect of IFN stimulation was not comparable with the effect observed in specific immune cells presented in kidney primary cell cultures and the hematopoietic tissues of the fish.

During evolution, some viruses have evolved sophisticated mechanisms to avoid the host innate immune system. In particular, some rhabdoviruses, such as human virus VSV (vesicular stomatitis virus) or RV (rabies virus), have developed counteractions to both IFN induction and IFN signaling [47]. These viruses have different mechanisms for antagonizing the type I interferon response and blocking the induction of antiviral molecules; however, in both cases, the objective is the evasion of the host immune defense [48]. In fish, the matrix protein of the novirhabdovirus, IHNV (Infectious hematopoietic necrosis virus), affects host cellular gene expression to inhibit the transcription of immune-related genes [49]; however, little is known about how this effect is orchestrated.

ZF4 cells and kidney primary cell cultures infected with SVCV did not show a typical anti-viral response upon IFN gene induction and the increased expression of ISGs, such as MX or IFITs [2], [50]. The blocking of the interferon system suggests that the virus suppresses the immune response in primary cell cultures.This response was also investigated when kidney cells were forced to mount an antiviral response through the stimulation with recombinant IFNsΦ and also were infected with the virus SVCV. In this case, the IFITs expression pattern was modulated, as described in human hepatocytes infected with hepatitis C virus [51]. Kidney cells treated with IFNΦ2 and 3 showed the reduced expression of almost all IFIT genes (IFIT12A made the difference), whereas cells treated with IFNΦ1 only showed the down modulation of IFIT17C after viral infection. This result could indicate that the virus avoids the host defense system and suppresses the expression of a specific subset of IFIT genes for the establishment of infection. The viral-mediated inhibition of the IFN system has been previously described [51], [52], [53]. The response pattern observed in cells treated with IFNΦ1 after SVCV infection might reflect different signaling pathways between cells stimulated with IFNsΦ from groups I and II. Importantly, IFIT12A was the only gene whose expression was synergistically induced through all interferons and in response to virus infection. The different behavior after *in vitro* stimulation together with the tissue-specific expression of IFITs genes suggests the expansion and differential functions of these genes.

However, when viral infection is conducted *in vivo*, after intraperitoneal infection, a clear up-regulation of the expression of IFNs and ISGs, including MXab and IFITs, was observed. The overall immunity of the host is required to orchestrate the effective control of viral infections, and the absence of a complete response often results in fatal infections [52]. Both ZF4 cells and primary cell head kidney leukocyte cultures exhibited limited defense against viral infections because of the incomplete host machinery and these models are, therefore, easily manipulated by SVCV. These cells respond to IFN stimulus but are unable to mount an effective response against virus.

The most induced IFITs after *in vivo* infection, 12B, 13A and 17A, were selected to confirm direct antiviral activity. The microinjection of zebrafish eggs at one-cell stage with expression plasmids encoding these genes induced a significant reduction in mortality after SVCV infection, highlighting the antiviral role that these proteins might play in non-mammalian species. In mammals, there is evidence implicating these proteins in the restriction of translation initiation through interactions with the translation initiation factor eIF-3 [44], [54]–[57]. Moreover, IFITs are able to sequester viral proteins, such as human papillomavirus helicase E1 [58] and inhibit virus replication through the direct binding and sequestering of viral nucleic acids [59]–[63]. However, it remains unknown whether the same mechanisms are also present in fish. We can confirm that the present results provide the basis for multiple future research studies concerning not only the protection of fish (particularly aquacultured species) against virus infections but also the investigation of the basic aspects of IFITs biology, which could be studied in zebrafish, an attractive model organism with numerous experimental advantages.

## Materials and Methods

### Sequence Retrieval and Analysis

The IFIT sequences were searched using the zebrafish genome assembly version Zv9 (www.ensembl.org/Daniorerio/), exploiting the conservation of synteny between the human and zebrafish genomes. The sequences were confirmed through PCR amplification using specific primers (Table S1) to obtain the full-length open reading frame (ORF) of each gene. The PCR products were subcloned into a pCR3.1 vector (Invitrogen) and transformed into One Shot TOP10F’ competent cells (Invitrogen) for subsequent sequencing and ORF confirmation.

The identity and similarity analysis between the zebrafish, human and mouse IFIT sequences was performed using MatGAT [64]. The TPR distribution was analyzed using TPRpred (http://tprpred.tuebingen.mpg.de/) [65], and the theoretical isoelectric point (pI) and the calculated molecular weight were determined using ExPaSy tools (http://us.expasy.org/tools). The 3D-structure of zebrafish IFITs was predicted using the I-TASSER server [66], selecting the model with the best C-score, and viewed through PyMOL (http://www.pymol.org). The Template Modeling Score (TM-score), a measure of structural similarity between two proteins, was also analyzed to identify structural analogs with known crystal architecture in the Protein Data Bank (PDB; http://www.rcsb.org/pdb/).

### Phylogenetic Tree and Analysis of Darwinian Selection

IFIT-family protein sequences were retrieved from the NCBI Protein, Uniprot and Ensembl databases based on annotation. The sequences were subsequently complemented using a blastp search for homologs in different databases. The initial sequence alignment was performed using the MAFFT online server following an E-INS-i strategy [67]. The resulting alignment was pruned using Gblocks 0.91b [68] and subsequently analyzed using ProtTest 3.2 [69] to determine the best-fit amino acid replacement model using the Akaike Information Criterion (AIC) [70], specified to estimate the maximum likelihood gene tree using PhyML 3.0 [71]. The nodal confidence was calculated using the aLRT method [72]. Edition and representation of the obtained tree was performed in FigTree v1.3.1 (http://tree.bio.ed.ac.uk/software/figtree/).

An estimation of the rates of synonymous (silent) and nonsynonymous (amino-acid-changing) substitutions was performed to identify a positive Darwinian selection in the zebrafish IFIT family using the PAML package version 4 [73]. The maximum-likelihood (ML) approach was implemented in the CODEML program to determine the ω value among zebrafish IFITs.

### Animals

Wild type adult zebrafish (*Danio rerio*) were grown in our experimental facilities according to established protocols [74], [75] (also see http://zfin.org/zf_info/zfbook/zfbk.html). Fish care and the challenge experiments were conducted according the CSIC National Committee on Bioethics under approval number 07_09032012.

### Cell Cultures and Viral Infection

Fibroblastic like cell line, ZF4, derived from 1-day-old zebrafish embryos (ATCC CRL-2050) [46] were cultured in Dulbecco’s modified Eagle’s medium (D/MEM/F12, Gibco) supplemented with 100 µg/mL of primocin (InvivoGen) and 10% fetal bovine serum (FBS) at 26°C. Human HEK-293 cells (ATCC CRL-1573) [76] were grown in Eagle's Minimum Essential Medium (Gibco) supplemented with 100 µg/mL primocin (InvivoGen), 1X non-essential amino acids (Gibco), 1 mM sodium pyruvate (Gibco) and 10% FBS. The cells were incubated in a 5% CO_2_ atmosphere at 37°C. Kidney cell suspensions were obtained from adult fish sacrificed using anaesthesia in ice. Kidneys were homogenized through a 100-µm mesh, and the mixture was adjusted to the required concentration (1.5×10^6^ cells/ml) in Leibovitz L-15 medium (Gibco) supplemented with 100 µg/mL of Primocin (InvivoGen) and 2% FBS and maintained at 26°C. For the *in vitro* stimulations, the cells were seeded into 24-well plates at 1 ml per well.

The rhabdovirus, spring viraemia of carp virus (SVCV isolate 56/70) was used in these experiments. Experimental infections were performed at 22°C, and the viral titer was calculated as previously described [77].

### RNA Extraction and Gene Expression

Total RNA isolation was performed using the Maxwell 16 LEV Simply RNA Tissue Kit (Promega, Madison, WI, USA) according to the manufacturer’s instructions. cDNA was obtained from 1 µg of total RNA using the SuperScript III First-Strand Synthesis Supermix (Invitrogen). Specific qPCR primers were designed (Table S1) using the *Primer3* program [78], and the primer efficiency was evaluated [79]. A previously described [80] cDNA template was used for real-time PCR amplification, with 40 cycles and a 60°C annealing temperature. All reactions were performed with several biological replicates and using technical triplicates. The relative expression levels of the genes were normalized to the expression of 18 S ribosomal RNA [81], as a housekeeping gene control (primers specified in Table S1), following the Pfaffl method [79].

### Production of Zebrafish Recombinant IFNsφ

Zebrafish IFNΦ1, IFNΦ2 and IFNΦ3 (GenBank accession numbers: NM_207640, NC_007114 and NC_007114, respectively) expression constructs in the pcDNA3.1/V5-His backbone were kindly provided by Dr. Mulero (University of Murcia, Spain). Recombinant IFNsΦ were produced by transfection of 6 µg the plasmids into HEK-293 cells at 70–80% confluence using the X-tremeGENE HP DNA Transfection Reagent (Roche) according to the manufacturer’s instructions. Forty-eight hours after transfection, the supernatants were collected and stored at −80°C until further use.

### Antiviral Activity of IFIT Genes in Zebrafish

Three selected IFITs (12B, 13A and 17A) were amplified using touchdown PCR (primers in Table S1), and the PCR products were cloned using the pcDNA 3.1/V5-His TOPO TA Expression Kit (Invitrogen). One Shot TOP10F competent cells (Invitrogen) were transformed to generate the plasmid constructs. Plasmid purifications were conducted using the PureLink HiPure Plasmid Midiprep Kit (Invitrogen). The recombinant plasmids were microinjected into one-cell stage zebrafish embryos with a glass microneedle using Narishige MN-151 micromanipulator and Narishige IM-30 microinjector. In each experiment, a total of 240 embryos were divided into 6 groups of 40 eggs (4 replicates of 10 embryos) and each batch was microinjected with the following treatments diluted in PBS: pcDNA 3.1-IFIT12B, pcDNA 3.1-IFIT13A, pcDNA 3.1-IFIT17A, pcDNA 3.1-empty, and PBS. An additional untreated group was included to control the egg quality and survival. The quantity of plasmid inoculated into each embryo was 100 pg/egg in a final volume of 2 nL. Three days after plasmid administration, the larvae were microinjected in the duct of Cuvier with 2 nL of a SVCV suspension at a final concentration of 10^3^ TCID_50_/ml. The mortalities due to the viral infection were registered for 3 days after infection before an independently feeding and therefore before an ethical approval is required (EU directive 2010_63) [82]. Fish condition was controlled three times a day. The viral transcription in IFIT-injected larvae was quantified through qPCR using specific primers for the N gene of the SVCV at 9 hours after the infection. The relative expression of the N gene was normalized to the expression of 18 S ribosomal RNA (primers specified in Table S1).

### Statistical Analysis

The results were expressed as the means ± standard error. The significant differences were determined using Students t-test. The data from the *in vivo* antiviral activity of IFITs was analyzed using one-way analysis of variance (ANOVA) followed by Tukeýs multiple comparison test.

## Supporting Information

Figure S1 **Constitutive expression of IFIT genes in ZF4 cells and in head kidney primary cell cultures.** The basal expression of the different IFIT genes was analyzed through real-time PCR in ZF4 cells and in leukocyte primary cell cultures from kidney. The relative expression level of the genes was normalized using the 18 S ribosomal RNA as a housekeeping gene. The graphs represent the mean ± standard error of three independent samples.
(TIF)

Figure S2 **Biological activity of recombinant zebrafish IFNs.** A. The biological activity of the supernatants from HEK-293 cells transfected with the expression plasmids of zf-IFNΦ1, zf-IFNΦ2 and zf-IFNΦ3 was measured in ZF4 cells dispensed in 96-well plates, treated for 2 h at 26°C with 100 µl of the supernatant containing one of the three different recombinant zf-IFNsΦ. After incubation, the spring viraemia of carp virus (SVCV) was titered. Supernatants obtained from HEK-293 cells transfected with an empty plasmid were used as control. The treatment of ZF4 cells with supernatants containing IFNΦ1, IFNΦ2 or IFNΦ3 induced a significant reduction of the viral titer (the infected cells treated with IFNΦ3 were those that showed the lowest viral titer. B. The treatment with the different zf-IFNsΦ induced a significant increase in MXab expression in both cell types at 4 and 24 hours. The results are represented as the mean ± standard error of three independent samples. The asterisk denotes significant differences with respect to the control cells (treated with supernatants obtained from HEK-293 cells transfected with the empty plasmid). Significant differences were displayed as ***(0.0001<p<0.001), **(0.001<p<0.01) or *(0.01<p<0.05).
(TIF)

Table S1 Sequence of specific primers designed for ORF confirmation, qPCR experiments and vector expression construction.
(XLSX)

Table S2 Accession numbers of the IFIT sequences obtained from GenBank, Ensembl and Uniprot databases used to conduct phylogenetic analyses.
(XLSX)

Table S3 **Identities and similarities.** Percentages of identity (grey) and similarity (white) between human/murine IFIT proteins and the 10 IFIT proteins identified in zebrafish.
(XLSX)

## References

[1] Kawai T, Akira S (2008) Toll-like Receptor and RIG-1-like Receptor Signaling. Ann NY Acad Sci 1143: 1–20. 19076341 10.1196/annals.1443.020

[2] Sadler AJ, Williams BR (2008) Interferon-inducible antiviral effectors. Nat Rev Immunol 8: 559–568. 18575461 10.1038/nri2314 PMC2522268

[3] Schoggins JW, Rice CM (2011) Interferon-stimulated genes and their antiviral effector functions. Curr Opin Virol 6: 519–525. 10.1016/j.coviro.2011.10.008 PMC3274382 22328912

[4] Zou J, Secombes CJ (2011) Teleost fish interferons and their role in immunity. Dev Comp Immunol 35: 1376–1387. 21781984 10.1016/j.dci.2011.07.001

[5] Robertsen B (2006) The interferon system of teleost fish. Fish Shellfish Immunol 20: 172–191. 15939626 10.1016/j.fsi.2005.01.010

[6] Stein C, Caccamo M, Laird G, Leptin M (2007) Conservation and divergence of gene families encoding components of innate immune response systems in zebrafish. Genome Biol 8: R251. 18039395 10.1186/gb-2007-8-11-r251 PMC2258186

[7] Zou J, Tafalla C, Truckle J, Secombes CJ (2007) Identification of a second group of type I IFNs in fish sheds light on IFN evolution in vertebrates. J Immunol 179: 3859–3871. 17785823 10.4049/jimmunol.179.6.3859

[8] Aggad D, Mazel M, Boudinot P, Mogensen KE, Hamming OJ, et al (2009) The two groups of zebrafish virus-induced interferons signal via distinct receptors with specific and shared chains. J Immunol 183: 3924–3931. 4343. 19717522 10.4049/jimmunol.0901495

[9] García MA, Gil J, Ventoso I, Guerra S, Domingo E, et al (2006) Impact of protein kinase PKR in cell biology: from antiviral to antiproliferative action. Microbiol Mol Biol Rev 70: 1032–1060. 17158706 10.1128/MMBR.00027-06 PMC1698511

[10] Martens S, Howard J (2006) The interferon-inducible GTPases. Annu Rev Cell Dev Biol 22: 559–589. 16824009 10.1146/annurev.cellbio.22.010305.104619

[11] Haller O, Staeheli P, Kochs G (2007) Interferon-induced Mx proteins in antiviral host defense. Biochimie 89: 812–818. 17570575 10.1016/j.biochi.2007.04.015

[12] Verrier ER, Langevin C, Benmansour A, Boudinot P (2011) Early antiviral response and virus-induced genes in fish. Dev Comp Immunol 35: 1204–1214. 21414349 10.1016/j.dci.2011.03.012

[13] Fensterl V, Wetzel JL, Ramachandran S, Ogino T, Stohlman SA, et al (2012) Interferon-induced Ifit2/ISG54 protects mice from lethal VSV neuropathogenesis. PLoS Pathog 8: e1002712. 22615570 10.1371/journal.ppat.1002712 PMC3355090

[14] Fensterl V, Sen GC (2011) The ISG56/IFIT1 gene family. J Interferon Cytokine Res 31: 71–78. 20950130 10.1089/jir.2010.0101 PMC3021354

[15] Diamond MS, Farzan M (2013) The broad-spectrum antiviral functions of IFIT and IFITM proteins. Nat Rev Immunol 13: 46–57. 23237964 10.1038/nri3344 PMC3773942

[16] Zhou X, Michal JJ, Zhang L, Ding B, Lunney JK, et al (2013) Interferon induced IFIT family genes in host antiviral defense. Int J Biol Sci 9: 200–208. 23459883 10.7150/ijbs.5613 PMC3584916

[17] D’Andrea LD, Regan L (2003) TPR proteins: the versatile helix. Trends Biochem Sci 28: 655–662. 14659697 10.1016/j.tibs.2003.10.007

[18] Zhu H, Cong JP, Shenk T (1997) Use of differential display analysis to assess the effect of human cytomegalovirus infection on the accumulation of cellular RNAs: induction of interferon-responsive RNAs. Proc Natl Acad Sci USA 94: 13985–13990. 9391139 10.1073/pnas.94.25.13985 PMC28419

[19] Saha S, Rangarajan PN (2003) Common host genes are activated in mouse brain by Japanese encephalitis and rabies viruses. J Gen Virol 84: 1729–1735. 12810866 10.1099/vir.0.18826-0

[20] Wacher C, Müller M, Hofer MJ, Getts DR, Zabaras R, et al (2007) Coordinated regulation and widespread cellular expression of interferon-stimulated genes (ISG) ISG-49, ISG-54, and ISG-56 in the central nervous system after infection with distinct viruses. J Virol 81: 860–871. 17079283 10.1128/JVI.01167-06 PMC1797448

[21] Rathi AV, Cantalupo PG, Sarkar SN, Pipas JM (2010) Induction of interferon-stimulated genes by Simian virus 40 T antigens. Virol 406: 202–211. 10.1016/j.virol.2010.07.018 PMC2939315 20692676

[22] Berchtold S, Manncke B, Klenk J, Geisel J, Autenrieth IB, et al (2008) Forced IFIT-2 expression represses LPS induced TNF-alpha expression at posttranscriptional levels. BMC Immunol 9: 75. 19108715 10.1186/1471-2172-9-75 PMC2632614

[23] Ovstebo R, Olstad OK, Brusletto B, Moller AS, Aase A, et al (2008) Identification of genes particularly sensitive to lipopolysaccharide (LPS) in human monocytes induced by wild-type versus LPS-deficient Neisseria meningitidis strains. Infect Immun 76: 2685–2695. 18362127 10.1128/IAI.01625-07 PMC2423066

[24] Kylaniemi MK, Haveri A, Vuola JM, Puolakkainen M, Lahesmaa R (2009) Gene expression signatures characterizing the development of lymphocyte response during experimental Chlamydia pneumoniae infection. Microb Pathog 46: 235–242. 19486640 10.1016/j.micpath.2009.01.006

[25] Liu Y, Zhang YB, Liu TK, Gui JF (2013) Lineage-specific expansion of IFIT gene family: an insight into coevolution with IFN gene family. PLoS One 8: e66859. 23818968 10.1371/journal.pone.0066859 PMC3688568

[26] Postlethwait JH, Woods IG, Ngo-Hazelett P, Yan YL, Kelly PD, et al (2000) Zebrafish comparative genomics and the origins of vertebrate chromosomes. Genome Res 10: 1890–1902. 11116085 10.1101/gr.164800

[27] Woods IG, Wilson C, Friedlander B, Chang P, Reyes DK, et al (2005) The zebrafish gene map defines ancestral vertebrate chromosomes. Genome Res 15: 1307–1314. 16109975 10.1101/gr.4134305 PMC1199546

[28] Soukup SW (1974) Evolution by gene duplication. S. Ohno, ed. Springer-Verlag, New York. 160 Teratology 9: 250–251.

[29] Ravi V, Bhatia S, Gautier P, Loosli F, Tay BH, et al (2013) Sequencing of Pax6 loci from the elephant shark reveals a family of Pax6 genes in vertebrate genomes, forged by ancient duplications and divergences. PLoS Genet 9: e1003177. 23359656 10.1371/journal.pgen.1003177 PMC3554528

[30] Kimura M (1977) Preponderance of synonymous changes as evidence for the neutral theory of molecular evolution. Nature 267: 275–276. 865622 10.1038/267275a0

[31] Yang Z, Bielawski JP (2000) Statistical methods for detecting molecular adaptation. Trends Ecol Evol 15: 496–503. 11114436 10.1016/S0169-5347(00)01994-7 PMC7134603

[32] Sunyer JO, Lambris JD (1998) Evolution and diversity of the complement system of poikilothermic vertebrates. Immunol Rev 166: 39–57. 9914901 10.1111/j.1600-065x.1998.tb01251.x

[33] Zhang J, Rosenberg HF, Nei M (1998) Positive Darwinian selection after gene duplication in primate ribonuclease genes. Proc Natl Acad Sci USA 95: 3708–3713. 9520431 10.1073/pnas.95.7.3708 PMC19901

[34] Vilches C, Parham P (2002) KIR: diverse, rapidly evolving receptors of innate and adaptative immunity. Annu Rev Immunol 20: 217–251. 11861603 10.1146/annurev.immunol.20.092501.134942

[35] Bustamante CD, Fledel-Alon A, Williamson S, Nielsen R, Hubisz MT, et al (2005) Natural selection on protein-coding genes in the human genome. Nature 437: 1153–1157. 16237444 10.1038/nature04240

[36] Viertlboeck BC, Habermann FA, Schmitt R, Groenen MA, Du Pasquier L, et al (2005) The chicken leukocyte receptor complex: a highly diverse multigene family encoding at least six structurally distinct receptor types. J Immunol 175: 385–393. 15972672 10.4049/jimmunol.175.1.385

[37] Yu XJ, Zheng HK, Wang J, Wang W, Su B (2006) Detecting lineage-specific adaptive evolution of brain-expressed genes in human using rhesus macaque as outgroup. Genomic 88: 745–751. 10.1016/j.ygeno.2006.05.008 16857340

[38] Stein C, Caccamo M, Laird G, Leptin M (2007) Conservation and divergence of gene families encoding components of innate immune response system in zebrafish. Genome Biol 8: R251. 18039395 10.1186/gb-2007-8-11-r251 PMC2258186

[39] Du Pasquier L, Wilson M, Sammut B (2009) The fate of duplicated immunity genes in the dodecaploid Xenopus ruwenzoriensis. Front Biosci 14: 177–191. 10.2741/3239 19273062

[40] Fernandes JMO, Ruangsri J, Kiron V (2010) Atlantic cod piscidin and its diversification through positive selection. PloS One 5: e9501. 20209162 10.1371/journal.pone.0009501 PMC2830478

[41] Boudinot P, van der Aa LM, Jouneau L, Du Pasquier L, Pontarotti P, et al (2011) Origin and Evolution of TRIM Proteins: New Insights from the Complete TRIM Repertoire of Zebrafish and Pufferfish. PLoS ONE 6: e22022. 21789205 10.1371/journal.pone.0022022 PMC3137616

[42] Daffis S, Samuel MA, Keller BC, Gale MJ, Diamond MS (2007) Cell-specific IRF-3 responses protect against West Nile virus infection by interferon-dependent and -independent mechanisms. PLoS Pathog 3: e106. 17676997 10.1371/journal.ppat.0030106 PMC1933455

[43] Fensterl V, White CL, Yamashita M, Sen GC (2008) Novel characteristics of the function and induction of murine p56 family proteins. J Virol 82: 11045–11053. 18768971 10.1128/JVI.01593-08 PMC2573266

[44] Terenzi F, White C, Pal S, Williams BR, Sen GC (2007) Tissue specific and inducer-specific differential induction of ISG56 and ISG54 in mice. J. Virol. 81: 8656–8665. 17553874 10.1128/JVI.00322-07 PMC1951374

[45] López-Muñoz A, Roca FJ, Meseguer J, Mulero V (2009) New insights into the evolution of IFNs: zebrafish group II IFNs induce a rapid and transient expression of IFN-dependent genes and display powerful antiviral activities. J Immunol 182: 3440–3449. 19265122 10.4049/jimmunol.0802528

[46] Driever W, Rangini Z (1993) Characterization of a cell line derived from zebrafish (Brachydanio rerio) embryos. In Vitro Cell Dev Biol Anim 29A: 749–754. 8407719 10.1007/BF02631432

[47] Rieder M, Conzelmann KK (2009) Rhabdovirus evasion of the interferon system. J Interferon Cytokine Res 29: 499–509. 19715459 10.1089/jir.2009.0068

[48] Faul EJ, Douglas SL, Schnell MJ (2009) Interferon response and viral evasion by members of the family Rhabdoviridae. Viruses 1: 832–851. 21994572 10.3390/v1030832 PMC3185512

[49] Chiou PP, Kim CH, Ormonde P, Leong JA (2000) Infectious hematopoietic necrosis virus matrix protein inhibits host-directed gene expression and induces morphological changes of apoptosis in cell cultures. J Virol 74: 7619–7627. 10906216 10.1128/jvi.74.16.7619-7627.2000 PMC112283

[50] Randall RE, Goodbourn S (2008) Interferons and viruses: an interplay between induction, signalling, antiviral responses and virus countermeasures. J Gen Virol 89: 1–47. 18089727 10.1099/vir.0.83391-0

[51] Raychoudhuri A, Shrivastava S, Steele R, Kim H, Ray R, et al (2011) ISG56 and IFITM1 proteins inhibit hepatitis C virus replication. J Virol 85: 12881–12889. 21976647 10.1128/JVI.05633-11 PMC3233139

[52] Samuel MA, Diamond MS (2006) Pathogenesis of West Nile Virus Infection: a Balance between Virulence, Innate and Adaptive Immunity, and Viral Evasion. J Virol 80: 9349–9360. 16973541 10.1128/JVI.01122-06 PMC1617273

[53] García-Sastre A (2011) Induction and evasion of type I interferon responses by influenza viruses. Virus Res 162: 12–18. 22027189 10.1016/j.virusres.2011.10.017 PMC3640439

[54] Guo J, Hui DJ, Merrick WC, Sen GC (2000) A new pathway of translational regulation mediated by eukaryotic initiation factor 3. EMBO J 19: 6891–6899. 11118224 10.1093/emboj/19.24.6891 PMC305884

[55] Guo J, Peters KL, Sen GC (2000) Induction of the human protein P56 by interferon, double-stranded RNA, or virus infection. Virology 267: 209–219. 10662616 10.1006/viro.1999.0135

[56] Hui DJ, Bhasker CR, Merrick WC, Sen GC (2003) Viral stress-inducible protein p56 inhibits translation by blocking the interaction of eIF3 with the ternary complex eIF2.GTP.Met-tRNAi. J Biol Chem 278: 39477–39482. 12885778 10.1074/jbc.M305038200

[57] Wang C, Pflugheber J, Sumpter RJ, Sodora DL, Hui D, et al (2003) Alpha interferon induces distinct translational control programs to suppress hepatitis C virus RNA replication. J Virol 77: 3898–3912. 12634350 10.1128/JVI.77.7.3898-3912.2003 PMC150642

[58] Terenzi F, Saikia P, Sen GC (2008) Interferon-inducible protein, P56, inhibits HPV DNA replication by binding to the viral protein E1. EMBO J 27: 3311–3321. 19008854 10.1038/emboj.2008.241 PMC2609736

[59] Daffis S, Szretter KJ, Schriewer J, Li J, Youn S, et al (2010) 2′-O methylation of the viral mRNA cap evades host restriction by IFIT family members. Nature 468: 452–456. 21085181 10.1038/nature09489 PMC3058805

[60] Pichlmair A, Lassnig C, Eberle CA, Górna MW, Baumann CL, et al (2011) IFIT1 is an antiviral protein that recognizes 5′-triphosphate RNA. Nat Immunol 12: 624–630. 21642987 10.1038/ni.2048

[61] Yang Z, Liang H, Zhou Q, Li Y, Chen H, et al (2012) Crystal structure of ISG54 reveals a novel RNA binding structure and potential functional mechanisms. Cell Res 22: 1328–1338. 22825553 10.1038/cr.2012.111 PMC3434343

[62] Abbas YM, Pichlmair A, Górna MW, Superti-Furga G, Nagar B (2013) Structural basis for viral 5′-PPP-RNA recognition by human IFIT proteins. Nature 494: 60–64. 23334420 10.1038/nature11783 PMC4931921

[63] Katibah GE, Lee HJ, Huizar JP, Vogan JM, Alber T, et al (2013) tRNA binding, structure, and localization of the human interferon-induced protein IFIT5. Mol Cell 49: 743–750. 23317505 10.1016/j.molcel.2012.12.015 PMC3615435

[64] Campanella JJ, Bitincka L, Smalley J (2003) MatGAT: an application that generates similarity/identity matrices using protein or DNA sequences. BMC Bioinform 4: 29. 10.1186/1471-2105-4-29 PMC166169 12854978

[65] Karpenahalli MR, Lupas AN, Söding J (2007) TPRpred: a tool for prediction of TPR-, PPR- and SEL1-like repeats from protein sequences. BMC Bioinformatics 8: 2. 17199898 10.1186/1471-2105-8-2 PMC1774580

[66] Roy A, Kucukural A, Zhang Y (2010) I-TASSER: a unified platform for automated protein structure and function prediction. Nat Protoc 5: 725–738. 20360767 10.1038/nprot.2010.5 PMC2849174

[67] Katoh K, Kuma K, Toh T, Miyata T (2005) MAFFT version 5: improvement in accuracy of multiple sequence alignment. Nucleic Acids Res 33: 511–518. 15661851 10.1093/nar/gki198 PMC548345

[68] Talavera G, Castresana J (2007) Improvement of phylogenies after removing divergent and ambiguously aligned blocks from protein sequence alignments. Syst Biol 56: 564–577. 17654362 10.1080/10635150701472164

[69] Darriba D, Taboada G, Doallo R, Posada D (2011) ProtTest 3: fast selection of best-fit models of protein evolution. Bioinformatics 27: 1164–1165. 21335321 10.1093/bioinformatics/btr088 PMC5215816

[70] Akaike H (1974) A new look at the statistical model identification. EEE Trans. Automatic Control Ac-19: 716–724.

[71] Guindon S, Dufayard JF, Lefort V, Anisimova M, Hordijk W, et al (2010) New algorithms and methods to estimate maximum-likelihood phylogenies: assessing the performance of PhyML 3.0. Syst Biol 59: 307–321. 20525638 10.1093/sysbio/syq010

[72] Anisimova M, Gascuel O (2006) Approximate Likelihood-Ratio Test for Branches: A Fast, Accurate, and Powerful Alternative. Syst Biol 55: 539–552. 16785212 10.1080/10635150600755453

[73] Yang Z (2007) PAML 4: phylogenetic analysis by maximum likelihood. Mol Biol Evol 24: 1586–1591. 17483113 10.1093/molbev/msm088

[74] Westerfield M (2000) The zebrafish book. A guide for the laboratory use of zebrafish (Danio rerio), 4th ed. University of Oregon Press, Eugene.

[75] Nusslein-Volhard C, Dahm R (2002) Zebrafish, a practical approach. Oxford University Press, Oxford.

[76] Graham FL, Smiley J, Russell WC, Nairn R (1977) Characteristics of a human cell line transformed by DNA from human adenovirus type 5. J Gen Virol 36: 59–74. 886304 10.1099/0022-1317-36-1-59

[77] Reed LJ, Muench H (1938) A simple method of estimating fifty percent endpoints. Am J Hyg 27: 493–497.

[78] Rozen S, Skaletsky H (2000) Primer3 on the WWW for general users and for biologist programmers. Methods Mol Biol 132: 365–386. 10547847 10.1385/1-59259-192-2:365

[79] Pfalffl MW (2001) A new mathematical model for relative quantification in real-time RT-PCR. Nucleic Acids Res 29: 2002–2007. 10.1093/nar/29.9.e45 PMC55695 11328886

[80] Díaz-Rosales P, Romero A, Balseiro P, Dios S, Novoa B, et al (2012) Microarray-based identification of differentially expressed genes in families of turbot (Scophthalmus maximus) after infection with viral haemorrhagic septicaemia virus (VHSV). Mar Biotechnol 14: 515–529. 22790792 10.1007/s10126-012-9465-0

[81] McCurley AT, Callard GV (2008) Characterization of housekeeping genes in zebrafish: male-female differences and effects of tissue type, developmental stage and chemical treatment. BMC Mol Biol 9: 102. 19014500 10.1186/1471-2199-9-102 PMC2588455

[82] Belanger SE, Balon EK, Rawlings JM (2010) Saltatory ontogeny of fishes and sensitive early life stages for ecotoxicology tests. Aquat. Toxicol 97: 88–95. 10.1016/j.aquatox.2009.11.020 20042243

