# Supplementary figures and images for: Interferon-Induced Genes of the Expanded IFIT Family Show Conserved Antiviral Activities in Non-Mammalian Species
Source: PLoS One. 2014 Jun 20;9(6):e100015. doi: 10.1371/journal.pone.0100015 (PMC4065003; doi:10.1371/journal.pone.0100015)

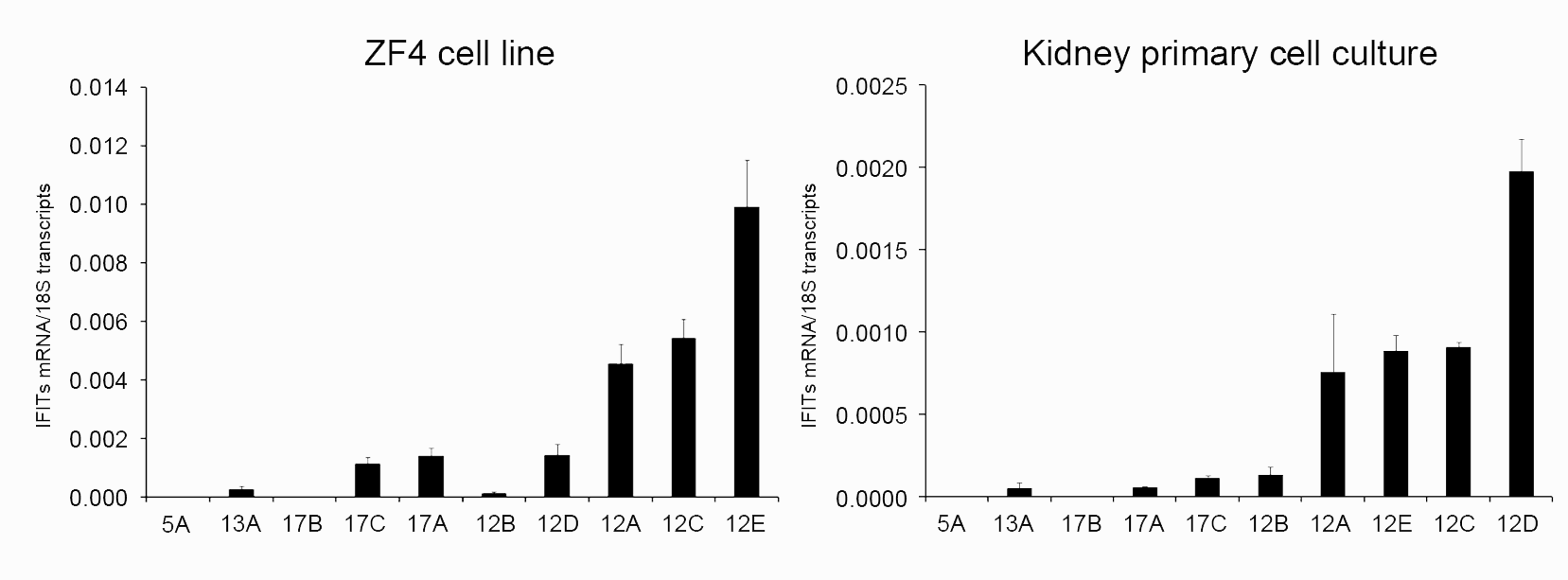

Supplement: Figure S1 — Constitutive expression of IFIT genes in ZF4 cells and in head kidney primary cell cultures. The basal expression of the different IFIT genes was analyzed through real-time PCR in ZF4 cells and in leukocyte primary cell cultures from kidney. The relative expression level of the genes was normalized using the 18 S ribosomal RNA as a housekeeping gene. The graphs represent the mean ± standard error of three independent samples. (TIF) [file pone.0100015.s001.tif]

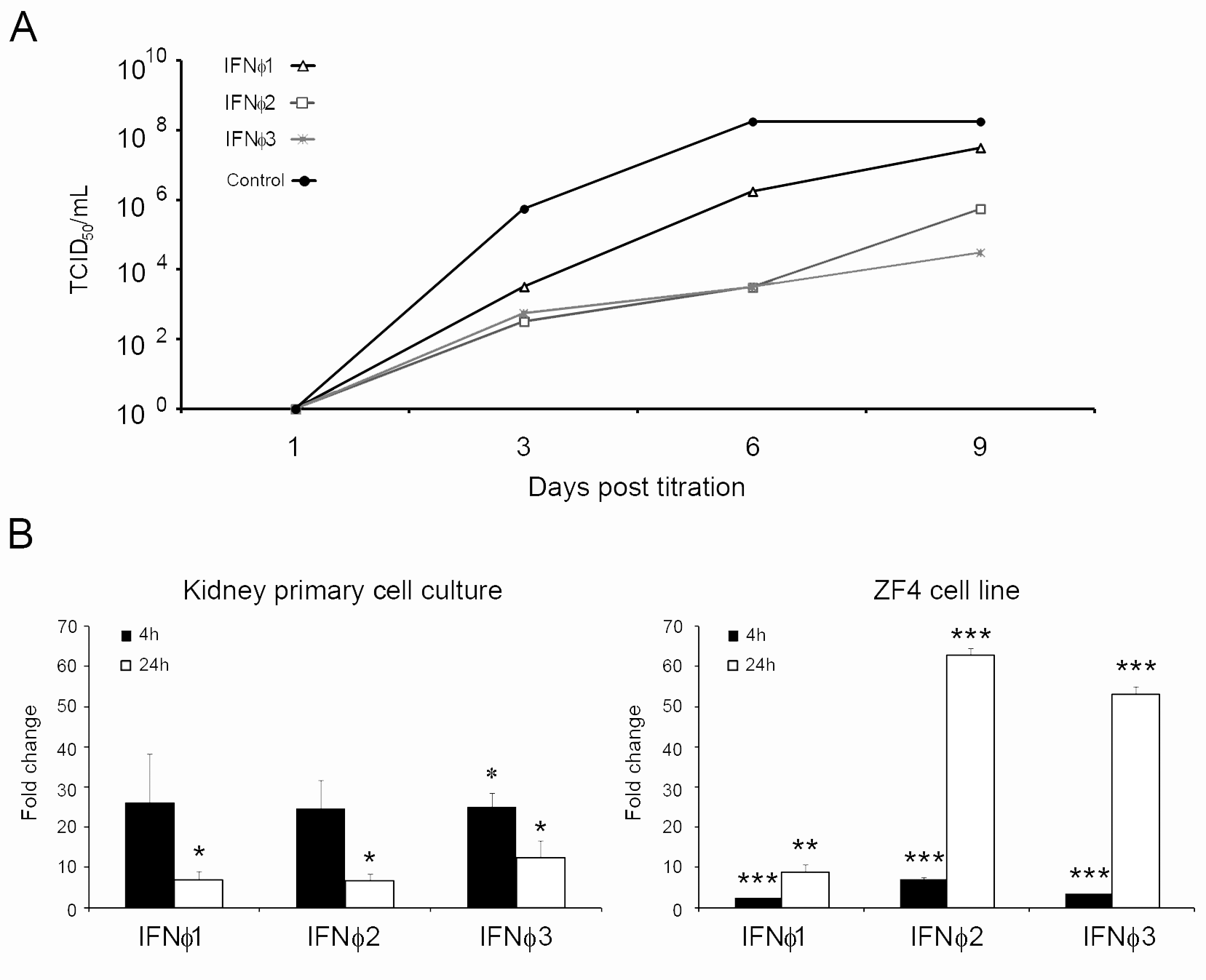

Supplement: Figure S2 — Biological activity of recombinant zebrafish IFNs. A. The biological activity of the supernatants from HEK-293 cells transfected with the expression plasmids of zf-IFNΦ1, zf-IFNΦ2 and zf-IFNΦ3 was measured in ZF4 cells dispensed in 96-well plates, treated for 2 h at 26°C with 100 µl of the supernatant containing one of the three different recombinant zf-IFNsΦ. After incubation, the spring viraemia of carp virus (SVCV) was titered. Supernatants obtained from HEK-293 cells transfected with an empty plasmid were used as control. The treatment of ZF4 cells with supernatants containing IFNΦ1, IFNΦ2 or IFNΦ3 induced a significant reduction of the viral titer (the infected cells treated with IFNΦ3 were those that showed the lowest viral titer. B. The treatment with the different zf-IFNsΦ induced a significant increase in MXab expression in both cell types at 4 and 24 hours. The results are represented as the mean ± standard error of three independent samples. The asterisk denotes significant differences with respect to the control cells (treated with supernatants obtained from HEK-293 cells transfected with the empty plasmid). Significant differences were displayed as ***(0.0001<p<0.001), **(0.001<p<0.01) or *(0.01<p<0.05). (TIF) [file pone.0100015.s002.tif]
